# Comparative Analysis of Texture Characteristics, Sensory Properties, and Volatile Components in Four Types of Marinated Tofu

**DOI:** 10.3390/foods13132068

**Published:** 2024-06-29

**Authors:** Bing Yang, Wanli Zhang, Heng Wang, Shenli Wang, Jing Yan, Zijie Dong, Penghui Zhao, Fazheng Ren, Lishui Chen

**Affiliations:** 1 Food Laboratory of Zhong Yuan, Luohe 462300, China; yangbing@zyfoodlab.com (B.Y.); renfazheng@263.net (F.R.); 2 School of Biological Engineering, Henan University of Technology, Zhengzhou 450001, China; 3 Key Laboratory of Precision Nutrition and Food Quality, Department of Nutrition and Health, China Agricultural University, Beijing 100193, China

**Keywords:** marinated tofu, HS-GC-IMS, key volatile flavor substance, ROAV, PLS-DA

## Abstract

In this study, three different brands of commercially available marinated tofu were analyzed and compared with homemade products to explore the effect of key flavor substances on their sensory quality, sensory properties, texture characteristics, and volatile components. The texture characteristics and flavor substances of the three brands of commercially available marinated tofu were significantly different from those of homemade products. A total of 64 volatile components were identified by headspace-gas chromatography-ion mobility spectrometry (HS-GC-IMS), mainly including 11 hydrocarbons, 11 alcohols, 10 ketones, 15 aldehydes, 4 esters, 1 acid, and 12 other volatile substances. Among these, nine key flavor compounds (ROAV > 1, VIP > 1) were identified using the relative odor activity value (ROAV) combined with a partial least squares discriminant analysis (PLS-DA) and variable importance in projection, including *α*-Pinene, *β*-Myrcene, *α*-Phellandrene, 1-Penten-3-one, Butanal, 3-Methyl butanal, acetic acid ethyl ester, 1,8-Cineol, and 2-Pentyl furan. The correlation heatmap showed that sensory evaluation was positively correlated with hardness, gumminess, chewiness, and springiness while negatively correlated with 2-Pentyl furan, *α*-Pinene, resilience, *α*-Phellandrene, 1-Penten-3-one, acetic acid ethyl ester, and 1,8-Cineol. Overall, this study provides a theoretical reference for developing new instant marinated tofu snacks.

## 1. Introduction

As one of the important crops in China, soybean contains many nutrients, such as proteins, vitamins, and minerals, and is an excellent resource of high-quality plant proteins [1]. Tofu (soybean curd), a traditional gourmet bean product, is a gel product with a three-dimensional network structure obtained by coagulating soybean proteins with coagulants. Studies have revealed that tofu could enhance immunity, prevent obesity, and reduce the risk of heart disease [2,3]. However, tofu is vulnerable to spoilage and has a short shelf life due to its high-water content and rich nutrients, which significantly limits its market recognition and the development of bean product processing industries [4]. Therefore, developing delicious, healthy, leisurely, and convenient instant marinated tofu products has garnered high importance. Similarly, marinated tofu, one of the representatives of tofu, must keep up with the trend and gradually be industrialized, standardized, and scaled up.

The production process of traditional tofu can be roughly divided into the following steps: soaking, grinding, filtering, heating of soymilk, adding coagulant, and pressing [5]. Raw materials, frying, marinating, sterilization, and other processes can also affect the production of volatile substances in products. The aroma of raw tofu is complex, mainly manifested in the aroma of fruit and oil, but also possesses a certain beany smell. The marinating process plays a vital role in seasoning and cooking during processing. Frying improves the texture of tofu and endows it with good color. However, the characteristic flavor substances of raw tofu may be lost post-high-temperature processing [6].

The flavor of marinated tofu determines the degree of consumer preference. Aroma is one of the important evaluation criteria for the quality and shape of products, directly affecting consumer preference and market recognition [7]. Therefore, analyzing the important flavor characteristic components of food is conducive to stabilizing and improving product quality. However, research on the aroma components of marinated tofu based on the difference in dietary preferences in China is scarce. Since not all flavor compounds can be perceived by human olfactory receptors, there are chances that they might affect the aroma characteristics. For many food products, only a limited number of key aroma-active compounds contribute to the overall aroma [8]. Thus, the quantitative verification of the roles of these key aroma-active compounds in the overall aroma of a wide range of commercial tofu is highly necessary. At present, the main methods for detecting volatile flavor components include gas chromatography-mass spectrometry (GC-MS) [9,10], gas chromatography-olfactometry-mass spectrometry (GC-O-MS) [11,12], electronic nose [13,14], gas chromatography-quadrupole time-of-flight mass spectrometry (GC/Q-TOF MS) [15], and full two-dimensional gas chromatography-time-of-flight mass spectrometry (GC × GC-TOFMS) [16]. Among them, GC-IMS technology has high sensitivity and provides a fast response, which is suitable for the separation and identification of trace substances, and thus, has been widely used in food industries [6].

In this study, different brands of marinated tofu and homemade tofu were selected as the research materials. The texture characteristics and sensory properties were evaluated by the sensory evaluation method and texture analyzer, respectively. The volatile substances were analyzed by the GC-IMS technology, and the key volatile components were further analyzed by PLS-DA. The key volatiles were identified using their relative odor activity values (ROAVs). The correlation among the volatile components, sensory evaluation, and texture parameters was explored, which provided a theoretical reference for the evaluation of the texture characteristics and index establishment of existing marinated tofu products.

## 2. Materials and Methods

### 2.1. Material and Reagents

Soybean and seasonings, including sparerib sauce, chicken sauce, yeast extract, salt, sugar, and flavored oil, were all purchased from local supermarkets. The commercially available tofu TF1, TF2, and TF3 were purchased from YOUNG SHINE KEE (YOUNG SHINE KEE Food Co., Ltd. Shanghai, Shanghai, China), Three Squirrels (Three Squirrels Co., Ltd., Hefei, China), and Gluttonous monkeys (Shanghai Golden Monkey Food Co., Ltd., Shanghai, China).

### 2.2. Sample Preparation

Three available types of marinated tofu were selected through preliminary investigation and combined with the sales ranking and favorable rate of mainstream e-commerce platforms, marked as TF1, TF2m and TF3. Each kind of independent prepackaged products with the same production date were randomly selected, and they were all spicy.

**Figure 1:**
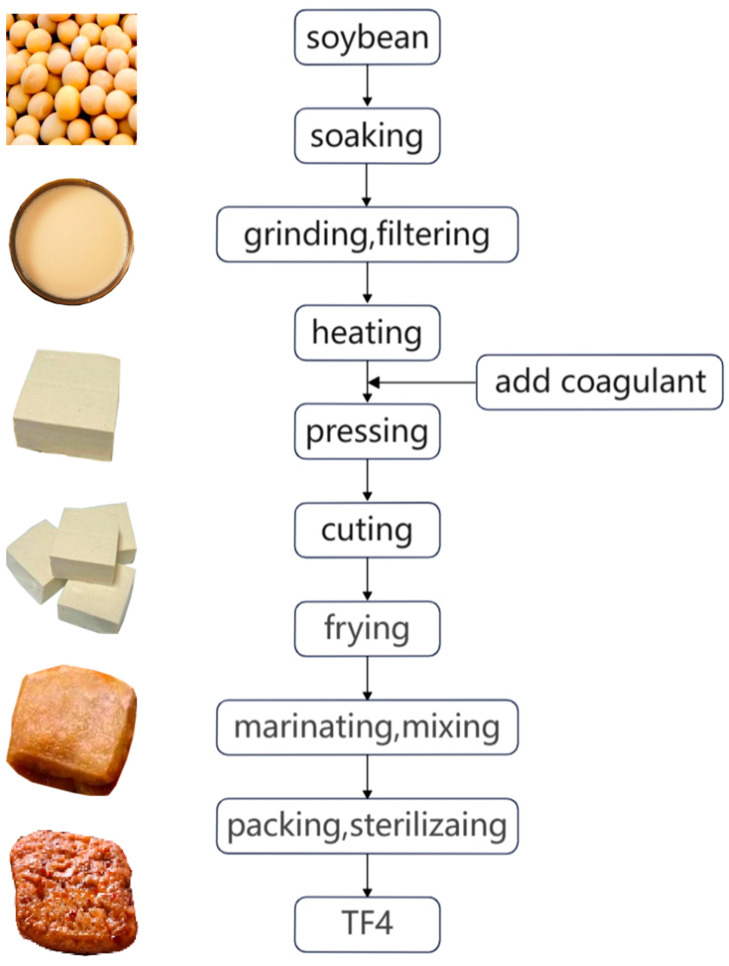
Process flow chart of TF4.

The process flow of the homemade samples (TF4) was as follows (Figure 1).

The soybeans were soaked in distilled water (*m*:*v* = 1:5), ground at 1:8 (*m:v*), and filtered to obtain raw soybean milk. Then, we heated the mixture to the boiling point for 3–5 min to obtain soybean milk, cooled it to 85 °C, and added the coagulant (MgCl_2_). We poured the mixture into the mold and pressed for 2 h. The tofu (4 × 4 × 1.5 cm) was fried twice (130–140 °C, 5 min, and 160–170 °C, 2–3 min) and marinated for 5 min. Then, the tofu was mixed with the flavored oil at 1:10 (*m:m*), sealed with a vacuum packaging bag, and sterilized at 115 °C for 20 min. 

### 2.3. Sensory Evaluation

Twelve volunteers with professional food background and no sensory defects were selected to form a sensory evaluation group to evaluate the color, taste, flavor, and texture of the different tofu. The total score was 100 points. The sensory evaluation criteria are shown in Table 1.

### 2.4. Textural Property Analysis (TPA)

The tofu was cut into 2 cm × 2 cm × 1 cm cubes and analyzed by a texture analyzer (CTX, Brookfield Inc., Middleboro, MA, USA). For the TPA, a stainless-steel P/36R cylindrical compression probe was selected. The measured parameters were: trigger type in AUTO (automatic), test rate of 1 mm/s, return rate of 1 mm/s, strain of 50%, and residence time between two compressors of 5 s.

### 2.5. Analysis of Volatile Compounds by HS–GC-IMS

The volatile compounds of the marinated tofu were analyzed by HS–GC-IMS (FlavourSpec, G.A.S., Dortmund, Germany). A sample of 2 g was placed into a headspace bottle and sealed. The test used an automated headspace sampling method with an injection volume of 500 µL, incubation time of 15 min, incubation temperature of 60 °C, injection needle temperature of 85 °C, and incubation speed of 500 r/min.

Using the MXT-WAX column (15 m × 0.53 mm × 1 µm), GC separation was performed at 60 °C. The programed flow of the carrier gas (nitrogen, 99.999% purity) was set as follows: the initial flow rate was 2 mL/min held for 2 min, then linearly increased to 10 mL/min within 8 min, and finally increased to 100 mL/min within 10 min and held for 10 min.

The IMS system was coupled with GC and maintained at 45 °C. The speed of the drift gas (nitrogen, 99.999% purity) was kept at 150 mL/min.

The data were collected and analyzed using the Vocal software (0.4.03). The application software includes built-in Reporter and Gallery Plot plug-ins for plotting two-dimensional and fingerprint chromatograms of volatile components. It uses n-ketones (C4~C9) as the external standard substance to calculate the retention index of volatile components. By comparing the mass spectra of the samples with those of the NIST 2020 (National Institute of Standards and Technology, Gaithersburg, MD, USA) and IMS mass spectrometry database, the identification of volatiles was achieved. The relative content of each volatile component was calculated using the peak area normalization method.

### 2.6. Calculation of Relative Odor Activity Values

The relative odor activity value is used to assess the contribution of each compound to the overall aroma of the sample.

The ROAV_stan_ of the compound with the largest contribution to the overall flavor of the sample is 100, and the ROAVs of the other components are calculated according to the following formula:

$$ROAV\approx \frac{C_{i}}{T_{i}}\times \frac{T_{stan}}{C_{stan}}\times 100$$

where *C_i_* is the relative content of a volatile compound, %; *T_i_* is the sensory threshold of a volatile compound, μg/kg; the relative content of volatile compounds with the largest contribution of *C_stan_* to flavor, %; and *T_stan_* is the sensory threshold of the volatile compounds that contribute the most to flavor, μg/kg.

### 2.7. Statistical Analysis

The results are expressed as the mean ± standard deviation. All data were analyzed using IBM SPSS Statistics 26.0 (SPSS Inc., Chicago, IL, USA) and a one-way analysis of variance (ANOVA), Duncan test (significance defined as *p* < 0.05), and correlation analysis were conducted. Origin 2018 (OriginLab Corporation, Northampton, MA, USA) was used for plots. Chemometrics was performed using SIMCA 14.1 (MKS Umetrics, Umea, Sweden).

## 3. Results and Discussion

### 3.1. Sensory Evaluation

The sensory scores of different brands of marinated tofu are shown in Table 2.

The sensory characteristics of TF1, TF2, and TF3 were similar, while TF4 was quite different from them (*p* < 0.05). The color of marinated tofu mainly depends on the amount of caramel added. A high amount of caramel addition changes the color of the tofu to yellowish brown; as TF4 had no caramel, its color was yellow or beige. 

The average sensory scores of TF1, TF2, and TF3 were 78.42, 81.33, and 79.58 points, respectively, among which the score of TF2 was the highest (81.33), indicating that the edible quality of TF2 has a certain potential.

### 3.2. Textural Property Analysis

Texture is the most direct index to reflect the changes in texture, structure, and tissue in any food and an important basis for judging the quality of food. The texture of tofu is the external representation of the protein’s three-dimensional network structure, and the strength of the three-dimensional network structure construction depends on the number of participating proteins and gel formation methods [17]. The texture characteristics of the samples were characterized by hardness, resilience, cohesiveness, springiness, gumminess, and chewiness. As shown in Table 3, the texture characteristics of the different brands of marinated tofu were significantly different. 

Hardness is a reflection of the degree to which a sample resists deformation, and the numerical value refers to the maximum pressure required to compress the sample [18]. The springiness refers to the height of the sample recovered within a specified time after being compressed to a specified deformation. The hardness, springiness, gumminess, and chewiness of TF4 were significantly higher than those of the other tofu products (*p* < 0.05). The hardness, springiness, gumminess, and chewiness of the different brands of commercially available tofu were 1375–1655 g, 3.60–4.90, 998–1280, and 4417–4809, respectively, showing relatively consistent texture characteristics. TF1 had the lowest hardness (1375 g) and gumminess (998), with no significant difference in chewiness among TF1, TF2, and TF3 (*p* > 0.05). Notably, TF4 was quite different from the commercially available marinated tofu in terms of the texture characteristics.

### 3.3. HS-GC-IMS Analysis

**Figure 2:**
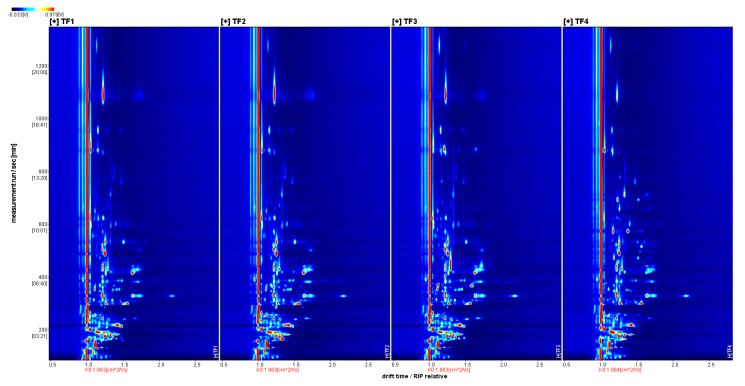
Two-dimensional GC-IMS spectrum of the samples.

**Figure 3:**
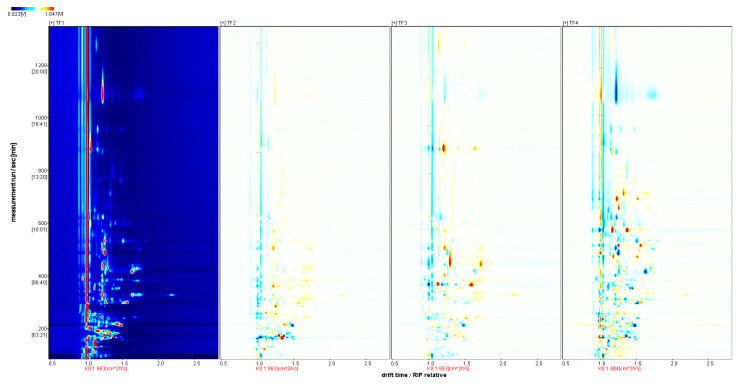
GC-IMS spectrum of the samples (difference diagram). Note: 1. The background of the spectrum is blue; the vertical coordinate represents the gas chromatographic retention time(s), and the horizontal coordinate represents the relative ion migration time. 2. The color represents the concentration of the substance; white means the concentration is low, and red means the concentration is high; the deeper the color, the greater the concentration.

**Figure 4:**
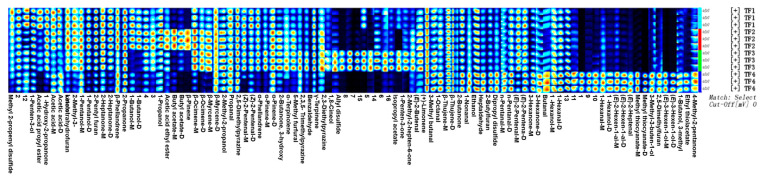
Gallery plot diagram of the samples of marinated tofu from different brands. Note: Each row represents all signal peaks selected in a sample, and every column represents the difference in signal peaks of the same substance in different samples; the darker the color, the higher the substance concentration and the stronger the signal peak.

As shown in Figure 2 and Figure 3, the blue area indicates that the substance was lower than that in TF1, whereas the red color indicates that it was higher than that in TF1; the deeper the color, the greater the difference. The volatile compositions of TF3 and TF4 showed significant differences from those of the other samples. The volatile components of marinated tofu were analyzed using HS-GC-IMS. Additionally, the crest volume of heterogeneous odor compounds on the fingerprint spectrum (Figure 4) was standardized to obtain an approximate percentage and explore the differences between marinated tofu products.The difference between TF1 and TF2 was relatively small, except for acetic acid ethyl ester, isopropyl acetate, acetic acid propyl ester, and butyl acetate. The contents of 5-Methylfurfural, 2,3-Diethylpyrazine, 1,8-Cineol, allyl disulfide, isopropyl acetate, 1-Pentene-3-one, etc., were in TF3. The content of 1-Hexanol, (*E*)-2-Hexen-1-al, (*E*)-2-Heptenal, 3-Methyl-3-buten-1-ol, and 2,5-Dimethyl furan were the highest in TF4 (Table 4).

**Figure 5:**
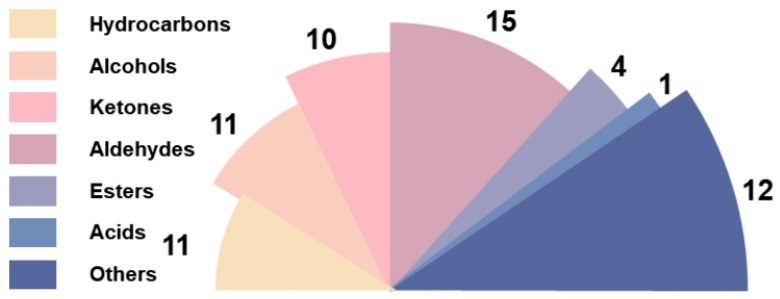
Species diagram of the volatile compounds of marinated tofu from different brands.

**Figure 6:**
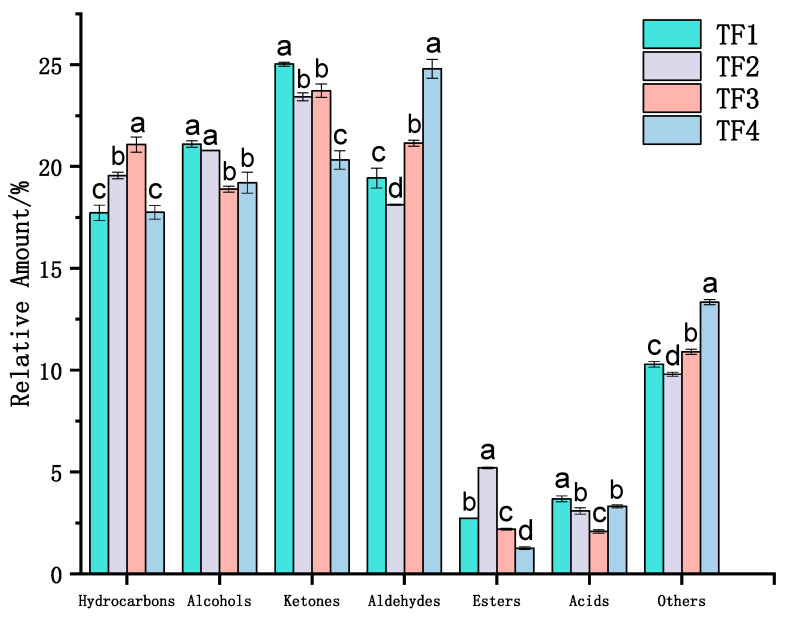
Relative content of the volatile compounds of marinated tofu from different brands. Different lowercase letters denote a significant difference (*p* < 0.05).

A total of 64 volatile substances were detected in four samples, including 11 hydrocarbons, 11 alcohols, 10 ketones, 15 aldehydes, 4 esters, 1 acid, and 12 other volatile substances (Figure 5). The results show that the volatile flavor compounds of the four marinated tofu were mainly composed of ketones, alcohols, aldehydes, and hydrocarbons, accounting for 20.32–25.03%, 18.89–21.10%, 18.13–24.80%, and 20.32–25.03%, respectively (Figure 6).

The mean and standard deviation of the relative content of each volatile compound are listed in Table 4. Studies have shown that 1-Hexanal, 1-Hexanol, 2-Pentyl furan, (*E*)-2-Hexen-1-al, acetic acid, and benzaldehyde contribute to the beany smell [19]. Alcohols significantly contribute to the aroma of soybean curd, and 1-Hexanol (pungent grass smell) in the raw material contributes to the beany smell, which comes from the enzymatic oxidation of linoleic acid and linolenic acid [20]. Linalool is an alcohol with the highest content and possesses a strong sweet floral fragrance. Ketones are important aroma substances in tofu, which are mainly produced by amino acid decomposition or the Maillard reaction during processing. 2-Heptanone contributes to a fruity flavor, similar to cinnamon in bean paste, soybean milk, and natto [6].

**Table 4:** Relative contents of the volatile compounds of marinated tofu from different brands.

Count	Compound Name	Formula	CAS	MW	RI	Rt [sec]	Relative Amount/%			
							TF1	TF2	TF3	TF4
Hydrocarbons (11)										
1	*α*-Pinene	C80568	C10H16	136.2	1025.5	244.293	1.46 ± 0.02 ^b^	1.86 ± 0.02 ^a^	1.91 ± 0.06 ^a^	1.60 ± 0.17 ^b^
2	*β*-Pinene	C127913	C10H16	136.2	1102.8	311.786	0.64 ± 0.03 ^c^	0.81 ± 0.01 ^b^	0.88 ± 0.01 ^a^	0.30 ± 0.03 ^d^
3	*β*-Thujene	C28634891	C10H16	136.2	1118.0	329.116	7.54 ± 0.16 ^b^	7.06 ± 0.04 ^c^	7.65 ± 0.09 ^b^	8.76 ± 0.14 ^a^
4	*δ*-3-Carene	C13466789	C10H16	136.2	1144.7	362.058	0.41 ± 0.00 ^c^	0.81 ± 0.03 ^a^	0.62 ± 0.00 ^b^	0.34 ± 0.02 ^d^
5	*β*-Myrcene	C123353	C10H16	136.2	1160.3	382.802	1.82 ± 0.06 ^c^	2.12 ± 0.04 ^b^	2.25 ± 0.06 ^a^	2.11 ± 0.08 ^b^
6	*α*-Phellandrene	C99832	C10H16	136.2	1173.3	400.953	0.78 ± 0.02 ^b^	0.84 ± 0.03 ^b^	1.41 ± 0.06 ^a^	0.38 ± 0.03 ^c^
7	(+)-Limonene	C138863	C10H16	136.2	1192.0	428.611	1.30 ± 0.03 ^b^	1.24 ± 0.02 ^c^	1.32 ± 0.01 ^b^	1.49 ± 0.01 ^a^
8	*β*-Phellandrene	C555102	C10H16	136.2	1201.9	443.305	1.53 ± 0.04 ^b^	1.56 ± 0.02 ^b^	1.28 ± 0.02 ^c^	1.69 ± 0.08 ^a^
9	*β*-Ocimene	C13877913	C10H16	136.2	1239.8	503.885	1.51 ± 0.06 ^c^	2.17 ± 0.03 ^b^	2.55 ± 0.04 ^a^	0.46 ± 0.03 ^d^
10	*γ*-Terpinene	C99854	C10H16	136.2	1250.1	521.526	0.33 ± 0.01 ^c^	0.40 ± 0.01 ^b^	0.48 ± 0.03 ^a^	0.32 ± 0.02 ^c^
11	*α*-Terpinolene	C586629	C10H16	136.2	1277.1	571.277	0.42 ± 0.01 ^c^	0.68 ± 0.01 ^b^	0.73 ± 0.02 ^a^	0.31 ± 0.01 ^d^
Total							17.73 ± 0.38 ^c^	19.56 ± 0.16 ^b^	21.08 ± 0.37 ^a^	17.76 ± 0.34 ^c^
Alcohols (11)										
12	2-Methyl-2-propanol	C75650	C4H10O	74.1	912.8	181.353	1.29 ± 0.01 ^b^	0.98 ± 0.02 ^c^	1.29 ± 0.01 ^b^	1.43 ± 0.05 ^a^
13	Ethanol	C64175	C2H6O	46.1	934.6	191.393	3.54 ± 0.34 ^b^	3.35 ± 0.03 ^b^	3.21 ± 0.14 ^b^	4.42 ± 0.2 ^a^
14	1-Propanol	C71238	C3H8O	60.1	1037.3	253.466	0.63 ± 0.03 ^b^	0.68 ± 0.01 ^a^	0.55 ± 0.03 ^c^	0.57 ± 0.00 ^c^
15	1- Butanol	C71363	C4H10O	74.1	1148.8	367.424	1.03 ± 0.05 ^a^	1.05 ± 0.01 ^a^	0.26 ± 0.00 ^c^	0.51 ± 0.03 ^b^
16	1-Penten-3-ol	C616251	C5H10O	86.1	1165.4	389.764	1.31 ± 0.03 ^a^	1.16 ± 0.02 ^b^	1.07 ± 0.07 ^b^	0.98 ± 0.05 ^c^
17	1-Butanol, 3-methyl	C123513	C5H12O	88.1	1211.4	457.634	0.38 ± 0.01 ^b^	0.38 ± 0.01 ^b^	0.29 ± 0.01 ^c^	0.78 ± 0.01 ^a^
18	1-Pentanol	C71410	C5H12O	88.1	1256.3	532.617	3.79 ± 0.19 ^b^	4.03 ± 0.04 ^a^	3.91 ± 0.07 ^ab^	2.69 ± 0.05 ^c^
19	3-Methyl-3-buten-1-ol	C763326	C5H10O	86.1	1260.8	540.729	0.07 ± 0.00 ^b^	0.07 ± 0.00 ^b^	0.07 ± 0.00 ^b^	0.21 ± 0.01 ^a^
20	(*E*)-3-Hexen-1-ol	C928972	C6H12O	100.2	1354.2	692.953	0.11 ± 0.01 ^b^	0.11 ± 0.01 ^b^	0.11 ± 0.00 ^b^	2.17 ± 0.14 ^a^
21	1 -Hexanol	C111273	C6H14O	102.2	1365.2	710.272	0.48 ± 0.07 ^c^	0.66 ± 0.08 ^b^	0.67 ± 0.01 ^b^	1.73 ± 0.07 ^a^
22	Linalool	C78706	C10H18O	154.3	1555.3	1087.114	8.46 ± 0.15 ^a^	8.33 ± 0.07 ^a^	7.45 ± 0.18 ^b^	3.71 ± 0.03 ^c^
Total							21.10 ± 0.16 ^a^	20.79 ± 0.01 ^a^	18.89 ± 0.15 ^b^	19.21 ± 0.51 ^b^
Ketones (10)										
23	2-Propanone	C67641	C3H6O	58.1	815.6	142.536	13.84 ± 0.09 ^a^	13.61 ± 0.07 ^a^	13.57 ± 0.09 ^a^	11.65 ± 0.24 ^b^
24	2-Butanone	C78933	C4H8O	72.1	898.2	174.898	4.17 ± 0.05 ^b^	3.23 ± 0.06 ^c^	3.38 ± 0.14 ^c^	4.76 ± 0.23 ^a^
25	4-Methyl-2-pentanone	C108101	C6H12O	100.2	1014.5	236.054	0.011 ± 0.00 ^c^	0.016 ± 0.00 ^b^	0.014 ± 0.00 ^b^	0.022 ± 0.00 ^a^
26	1-Penten-3-one	C1629589	C5H8O	84.1	1015.1	236.543	0.21 ± 0.02 ^b^	0.21 ± 0.00 ^b^	0.51 ± 0.02 ^a^	0.07 ± 0.00 ^c^
27	3-Hexanone	C589388	C6H12O	100.2	1096.6	304.963	0.53 ± 0.01 ^b^	0.40 ± 0.01 ^c^	0.50 ± 0.01 ^b^	0.93 ± 0.03 ^a^
28	2-Heptanone	C110430	C7H14O	114.2	1183.4	415.646	4.28 ± 0.09 ^a^	3.99 ± 0.07 ^b^	3.62 ± 0.16 ^c^	1.91 ± 0.02 ^d^
29	2-Methyl-3-ketotetrahydrofuran	C3188009	C5H8O2	100.1	1269.6	556.989	0.37 ± 0.02 ^a^	0.34 ± 0.01 ^b^	0.35 ± 0.02 ^ab^	0.19 ± 0.00 ^c^
30	2-Butanone, 3-hydroxy	C513860	C4H8O2	88.1	1289.2	595.229	0.35 ± 0.06 ^b^	0.38 ± 0.02 ^b^	0.52 ± 0.04 ^a^	0.23 ± 0.12 ^c^
31	1-Hydroxy-2-propanone	C116096	C3H6O2	74.1	1307.2	623.804	1.20 ± 0.04 ^a^	1.11 ± 0.03 ^a^	1.06 ± 0.04 ^a^	0.46 ± 0.21 ^b^
32	2-Methyl-2-hepten-6-one	C110930	C8H14O	126.2	1346.8	681.715	0.08 ± 0.00 ^c^	0.14 ± 0.01 ^b^	0.18 ± 0.01 ^a^	0.09 ± 0.00 ^c^
Total							25.03 ± 0.10 ^a^	23.43 ± 0.19 ^b^	23.72 ± 0.33 ^b^	20.32 ± 0.46 ^c^
Aldehydes (15)										
33	Propanal	C123386	C3H6O	58.1	778.5	130.069	1.87 ± 0.03 ^ab^	1.82 ± 0.04 ^b^	1.92 ± 0.04 ^a^	1.91 ± 0.07 ^ab^
34	Butanal	C123728	C4H8O	72.1	868.2	162.380	0.15 ± 0.01 ^b^	0.05 ± 0.00 ^c^	0.13 ± 0.00 ^b^	0.21 ± 0.03 ^a^
35	3-Methyl butanal	C590863	C5H10O	86.1	912.8	181.353	1.31 ± 0.04 ^b^	0.91 ± 0.05 ^c^	1.45 ± 0.05 ^a^	1.47 ± 0.09 ^a^
36	n-Pentanal	C110623	C5H10O	86.1	986.6	217.707	3.51 ± 0.13 ^b^	3.20 ± 0.02 ^c^	3.43 ± 0.04 ^b^	4.59 ± 0.11 ^a^
37	(*E*)-2-Butenal	C123739	C4H6O	70.1	1051.3	264.774	0.10 ± 0.00 ^b^	0.11 ± 0.00 ^a^	0.12 ± 0.00 ^a^	0.08 ± 0.00 ^c^
38	1-Hexanal	C66251	C6H12O	100.2	1091.2	299.928	4.29 ± 0.09 ^b^	4.33 ± 0.04 ^b^	4.31 ± 0.06 ^b^	6.13 ± 0.08 ^a^
39	(*Z*)-2-Pentenal	C1576869	C5H8O	84.1	1105.3	314.522	1.38 ± 0.03 ^a^	1.11 ± 0.03 ^b^	1.35 ± 0.01 ^a^	0.57 ± 0.02 ^c^
40	(*E*)-2-Pentenal	C1576870	C5H8O	84.1	1136.4	351.462	1.85 ± 0.04 ^b^	1.70 ± 0.01 ^c^	2.08 ± 0.01 ^a^	1.32 ± 0.02 ^d^
41	Heptaldehyde	C111717	C7H14O	114.2	1185.7	419.103	0.31 ± 0.01 ^b^	0.31 ± 0.00 ^b^	0.34 ± 0.01 ^b^	0.72 ± 0.05 ^a^
42	(*E*)-2-Hexen-1-al	C6728263	C6H10O	98.1	1220.6	472.090	0.39 ± 0.03 ^c^	0.47 ± 0.02 ^b^	0.34 ± 0.03 ^c^	1.89 ± 0.06 ^a^
43	1-Octanal	C124130	C8H16O	128.2	1291.7	600.271	0.47 ± 0.06 ^c^	0.57 ± 0.02 ^b^	0.51 ± 0.00 ^bc^	0.81 ± 0.05 ^a^
44	(*E*)-2-Heptenal	C18829555	C7H12O	112.2	1331.3	658.340	0.13 ± 0.01 ^b^	0.16 ± 0.01 ^b^	0.14 ± 0.01 ^b^	1.09 ± 0.08 ^a^
45	1-Nonanal	C124196	C9H18O	142.2	1396.6	762.009	0.31 ± 0.02 ^b^	0.33 ± 0.01 ^b^	0.33 ± 0.01 ^b^	0.55 ± 0.03 ^a^
46	Benzaldehyde	C100527	C7H6O	106.1	1499.3	958.971	1.86 ± 0.07 ^b^	1.76 ± 0.01 ^b^	2.06 ± 0.05 ^a^	1.35 ± 0.05 ^c^
47	5-Methyl furfural	C620020	C6H6O2	110.1	1628.0	1279.329	1.51 ± 0.05 ^c^	1.30 ± 0.02 ^c^	2.62 ± 0.17 ^a^	2.12 ± 0.15 ^b^
Total							19.44 ± 0.49 ^c^	18.13 ± 0.03 ^d^	21.14 ± 0.15 ^b^	24.80 ± 0.46 ^a^
Esters (4)										
48	Acetic acid ethyl ester	C141786	C4H8O2	88.1	875.6	165.385	0.66 ± 0.01 ^c^	4.00 ± 0.04 ^a^	0.67 ± 0.03 ^c^	0.96 ± 0.05 ^b^
49	Isopropyl acetate	C108214	C5H10O2	102.1	882.3	168.143	0.20 ± 0.00 ^b^	0.02 ± 0.01 ^c^	0.41 ± 0.03 ^a^	0.02 ± 0.00 ^c^
50	Acetic acid propyl ester	C109604	C5H10O2	102.1	975.8	211.946	1.69 ± 0.02 ^a^	0.52 ± 0.01 ^c^	0.85 ± 0.05 ^b^	0.21 ± 0.01 ^d^
51	Butyl acetate	C123864	C6H12O2	116.2	1074.7	284.879	0.18 ± 0.00 ^c^	0.67 ± 0.01 ^a^	0.25 ± 0.01 ^b^	0.06 ± 0.00 ^d^
Total							2.73 ± 0.01 ^b^	5.20 ± 0.04 ^a^	2.19 ± 0.05 ^c^	1.25 ± 0.07 ^d^
Acids (1)										
52	Acetic acid	C64197	C2H4O2	60.1	1463.1	884.22	3.68 ± 0.15 ^a^	3.09 ± 0.16 ^b^	2.08 ± 0.09 ^c^	3.31 ± 0.08 ^b^
Total							3.68 ± 0.15 ^a^	3.09 ± 0.16 ^b^	2.08 ± 0.09 ^c^	3.31 ± 0.08 ^b^
Others (12)										
53	2,5-Dimethylfuran	C625865	C6H8O	96.1	958.1	202.849	0.05 ± 0.02 ^b^	0.05 ± 0.00 ^b^	0.06 ± 0.00 ^b^	0.18 ± 0.01 ^a^
54	Ethyl thiolacetate	C625605	C4H8OS	104.2	1109.8	319.571	0.03 ± 0.01 ^b^	0.02 ± 0.00 ^b^	0.02 ± 0.00 ^b^	0.12 ± 0.01 ^a^
55	2-Butylfuran	C4466244	C8H12O	124.2	1132	345.990	0.41 ± 0.01 ^b^	0.34 ± 0.01 ^c^	0.40 ± 0.01 ^b^	0.74 ± 0.02 ^a^
56	1,8-Cineol	C470826	C10H18O	154.3	1203.3	445.292	0.27 ± 0.01 ^c^	0.32 ± 0.02 ^bc^	1.06 ± 0.04 ^a^	0.33 ± 0.03 ^b^
57	2-Pentyl furan	C3777693	C9H14O	138.2	1230.3	487.817	7.68 ± 0.09 ^a^	7.22 ± 0.10 ^b^	6.35 ± 0.17 ^c^	4.74 ± 0.06 ^d^
58	Methyl thiocyanate	C556649	C2H3NS	73.1	1277.4	571.914	0.25 ± 0.01 ^b^	0.31 ± 0.02 ^b^	0.33 ± 0.02 ^b^	6.21 ± 0.08 ^a^
59	Methyl 2-propenyl disulfide	C2179580	C4H8S2	120.2	1304.2	619.601	0.30 ± 0.04 ^a^	0.25 ± 0.02 ^a^	0.24 ± 0.01 ^a^	0.15 ± 0.06 ^b^
60	2,5-Dimethylpyrazine	C123320	C6H8N2	108.1	1326.1	650.697	0.52 ± 0.06 ^a^	0.58 ± 0.01 ^a^	0.65 ± 0.02 ^a^	0.16 ± 0.12 ^b^
61	2,3,5- Trimethylpyrazine	C14667551	C7H10N2	122.2	1396.6	762.009	0.21 ± 0.01 ^b^	0.19 ± 0 ^bc^	0.37 ± 0.04 ^a^	0.17 ± 0.02 ^c^
62	Dipropyl disulfide	C629196	C6H14S2	150.3	1406.9	779.807	0.16 ± 0.02 ^c^	0.19 ± 0.01 ^b^	0.16 ± 0.01 ^c^	0.37 ± 0.01 ^a^
63	2,3-Diethylpyrazine	C15707241	C8H12N2	136.2	1456.4	871.169	0.36 ± 0.03 ^b^	0.31 ± 0 ^b^	0.74 ± 0.06 ^a^	0.11 ± 0.02 ^c^
64	Allyl disulfide	C2179579	C6H10S2	146.3	1464.3	886.593	0.05 ± 0.01 ^b^	0.05 ± 0 ^b^	0.52 ± 0.07 ^a^	0.06 ± 0.01 ^b^
Total							10.29 ± 0.14	9.80 ± 0.1 ^d^	10.90 ± 0.13 ^b^	13.34 ± 0.13 ^a^

Note: Different letters in the same row indicate a statistically significant difference (*p* < 0.05).

Aldehydes are mainly produced by the catalytic decomposition of linoleic acid and linolenic acid by lipoxygenase in soybean. The content of 1-Hexanal was the highest in aldehydes, which is the main compound related to the beany smell [21]. (*E*)-2-Heptenal in TF4 was significantly higher than in the other groups, which enhances the fruit flavor. 1-Nonanal has a floral, waxy aroma, whereas n-Pentanal contributes to a pungent, fruity odor [22].

Esters are mainly formed by the esterification of organic acids and alcohols and provide a pleasant fruit aroma [23]. For instance, ethyl acetate provides a pleasant ethereal and fruity aroma. Furan compounds include 2-Pentyl furan, 2-Butylfuran, and 2,5-Dimethylfuran, which come from the Maillard reaction and caramelization reaction [4]. The high content of 2-Pentylfuran in the four samples endowed the final product with a pleasant flavor. Studies have shown that the aroma threshold of hydrocarbon compounds is higher, and their contribution to the overall flavor is lower [24,25].

### 3.4. Relative Calculation of Odor Activity Values 

**Figure 7:**
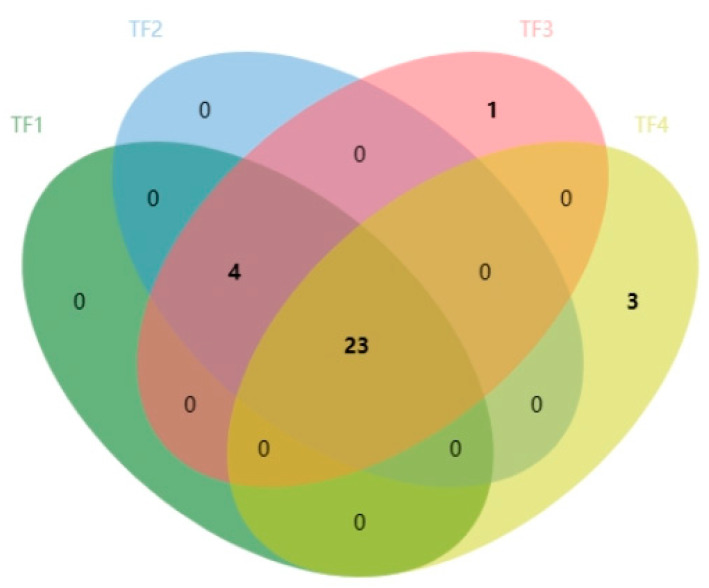
Venn diagram of volatile compounds with a ROAV ≥ 1 of marinated tofu from different brands.

The final contribution of a specific compound to the overall aroma of a sample depends on its concentration and odor threshold measured by the odor activity value (ROAV) [26] (Table 5). The odor threshold refers to the lowest concentration of a certain aroma substance. The greater the ROAV, the greater the contribution of the compound to the overall flavor. Typically, a component with a greater ROAV value is considered the main contributor to the overall aroma of a sample [27]. In this study, a total of 31 volatile flavor compounds were detected by evaluating ROAV > 1 compounds in marinated tofu, including 23 volatile compounds in the four samples and 27 volatile compounds in TF1, TF2, and TF3. The 23 common volatile compounds were *α*-Pinene, *β*-Myrcene, (+)-Limonene, *β*-Phellandrene, 1-Butanol, 3-Methyl, 1-Pentanol, 1 -Hexanol, linalool, propanal, butanal, 3-Methyl butanal, n-Pentanal, (*E*)-2-Butenal, 1-Hexanal, heptaldehyde, (*E*)-2-Hexen-1-al, 1-Octanal, 1-Nonanal, acetic acid ethyl ester, 2-Butylfuran, 1,8-Cineol, 2-Pentyl furan, and methyl thiocyanate. 1-Penten-3-one only appeared in TF3, while (*E*)-3-Hexen-1-ol, 3-Hexanone, and (*E*)-2-Heptenal only appeared in TF4 (Figure 7 and Table 5). *β*-Myrcene, contributing to the spicy flavor, had the highest ROAV of 100, making a substantial contribution to the overall flavor. Simultaneously, linalool, 3-Methyl butanal, 1-Hexanal, 1-Octanal, and 2-Pentyl furan, with a ROAV ≥ 30, significantly influenced the overall flavor profile. The contents of monoterpenoids, such as (+)-Limonene, *β*-Myrcene, *α*-Phellandrene, and linalool, were relatively high in the samples, suggesting that they are the key volatile compounds in Zanthoxylum bungeanum [28].

### 3.5. PLS-DA and Model Evaluation Analysis

**Figure 8:**
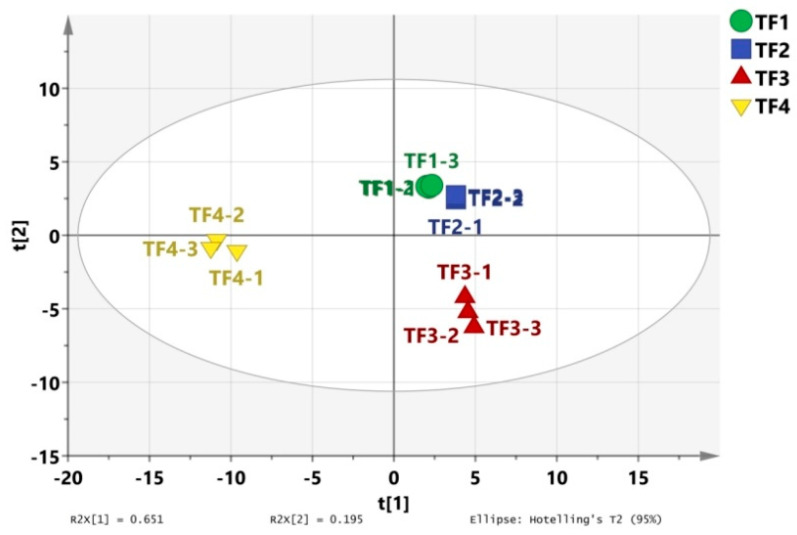
Scattering conditions of the PLS-DA score of the volatile compounds of marinated tofu from different brands.

**Figure 9:**
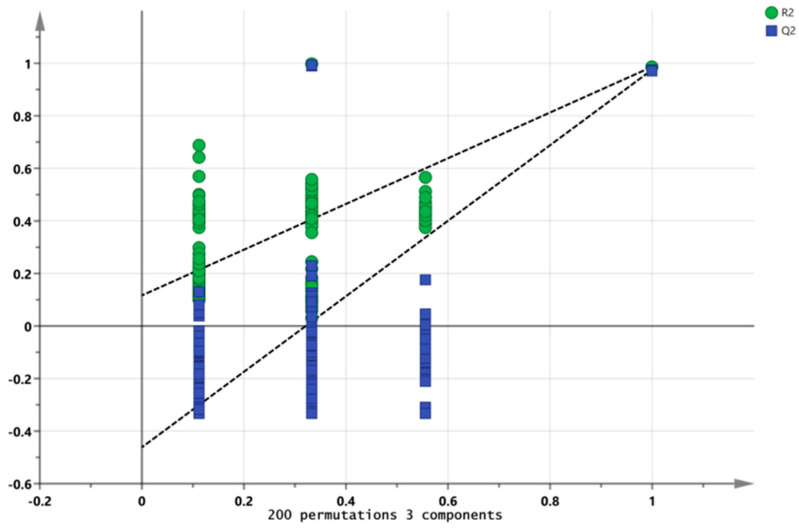
Permutation test of the volatile compounds of marinated tofu from different brands.

A partial least squares discriminant analysis (PLS-DA) can extract effective information about volatile compounds and reflect the differences in volatile substances among different samples. It is widely used to analyze the volatile substance characteristics of samples and is suitable for a situation with a large number of explanatory variables. R2X and R2Y stand for the explanation rate of the simulation to X and Y vectors, respectively, and Q^2^ denotes the prediction ability in the actual simulation. The parameters R^2^ and Q^2^, with values higher than 0.5 and near 1.0, were deemed as exact outcomes [29]. The results show that the independent variable fitting index (R 2X) was 0.964, the dependent variable fitting index (R 2Y) was 0.991, and the model prediction index (Q^2^) was 0.984, with R^2^ and Q^2^ exceeding 0.5, indicating that the model fit results were acceptable (Figure 8). The model Q^2^ of 200 permutation tests = −0.462, indicating that there was no overfitting phenomenon in the model, and the results are relatively reliable (Figure 9).

### 3.6. Key Volatile Substances

**Figure 10:**
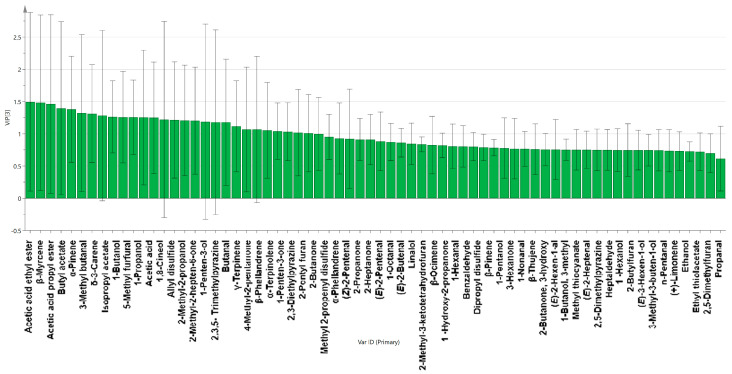
VIP value of the volatile flavor substances.

After detecting 64 odorant compounds in the four marinated tofu samples using GC-IMS, the VIP values obtained through a reliable PLS-DA matching model were used to evaluate the contribution of each odor compound to the overall aroma of marinated tofu. The weight of the PLS-DA model variables was measured using the variable importance for the projection (VIP). The greater the VIP value, the greater the contribution rate of volatile compounds to the overall flavor of marinated tofu. Flavor components with VIP values above 1.0 are considered discriminative indicator chemicals in many food specimens [30]. As shown in Figure 10, 26 volatile substances screened by PLS-DA significantly contributed to the flavor of marinated tofu (*p* < 0.05, VIP > 1). The key volatile substances of marinated tofu were identified through a comprehensive analysis with the ROAV value (VIP > 1, ROAV > 1), and the substances were as follows: *α*-Pinene, *β*-Myrcene, *α*-Phellandrene, 1-Penten-3-one, butanal, 3-Methyl butanal, acetic acid ethyl ester, 1,8-Cineol, and 2-Pentyl furan.

### 3.7. Correlation Analysis

The four marinated tofu samples were classified according to different perspectives using sensory evaluation, texture characteristics, and GC–IMS. The correlation heatmap between the key aroma-active compounds, texture, and sensory evaluation displayed a potential relationship among them. 

**Figure 11:**
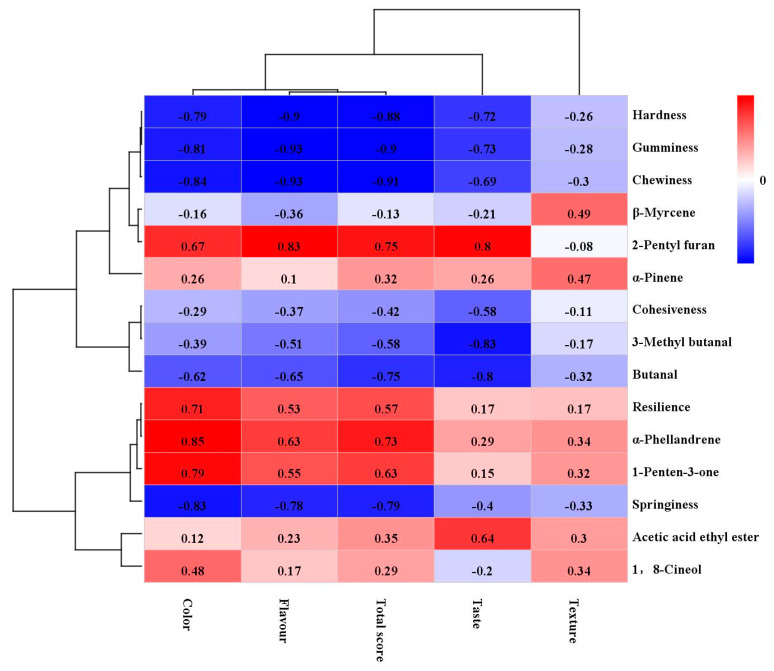
Pearson’s correlation heatmap displays the correlation between texture, volatile substances, and sensory evaluation of marinated tofu from different brands. Note: Each color represents the correlation coefficient, with blue and red indicating negative and positive correlations, respectively.

As shown in Figure 11, the color, flavor, total score, and taste were positively associated with 2-Pentyl furan, *α*-Pinene, resilience, *α*-Phellandrene, 1-Penten-3-one, acetic acid ethyl ester, and 1,8-Cineol but negatively associated with hardness, gumminess, chewiness, springiness, 3-Methyl butanal, and butanal. There was no significant correlation between the texture and various indicators (*p* > 0.05).

## 4. Conclusions

In this study, the volatile substances, texture characteristics, and sensory properties of three different brands of commercially available marinated tofu were investigated. The detection indexes of TF1, TF2, and TF3 were similar, and TF4 was significantly different from the commercially available marinated tofu in all aspects. A total of 64 volatile aroma components were detected, among which 9 key aroma components were identified (VIP > 1, ROAV > 1), including *α*-Pinene, *β*-Myrcene, *α*-Phellandrene, 1-Penten-3-one, butanal, 3-Methyl butanal, acetic acid ethyl ester, 1,8-Cineol, and 2-Pentyl furan. Additionally, the overall aroma difference in these different types of marinated tofu was higher due to the quantity of these compounds in their composition. 

The differentiation in aroma compounds among the different types of marinated tofu was assessed by PLS-DA. Pearson’s correlation analyses revealed distinct correlations between sensory evaluation, texture characteristics, and key aroma-active compounds. In summary, these results provide valuable information for the marinated tofu industry. Nevertheless, the interaction between the aroma compounds and food matrix needs further exploration to clarify the influence of processing and ingredients on the flavor and quality of tofu.

## Figures and Tables

**Table 1:** Sensory evaluation criteria of marinated tofu from different brands.

Items	Scoring Criteria	Score
Color (25%)	The epidermis is yellow-brown, uniform, and shiny.	20–25
	The epidermis is yellow-brown, relatively uniform, and shiny.	14–19
	The epidermis is uneven and dull.	7–13
	Too dark or too light in color, dull.	0–6
Taste (25%)	The taste is delicate, tender, juicy, and chewy.	20–25
	The taste is delicate, delicious, and chewy.	14–19
	The taste is acceptable and the chewiness is average.	7–13
	Rough taste and poor chewiness.	0–6
Flavour (25%)	It has a strong tofu flavor, strong spicy taste, and harmonious taste.	20–25
	It has a certain tofu flavor and moderate seasoning.	14–19
	General flavor, uncoordinated seasoning.	7–13
	Poor flavor, insufficient aroma.	0–6
Texture (25%)	Moderate hardness, good elasticity, and complete shape.	20–25
	General hardness, general elasticity, and relatively complete shape.	14–19
	Poor hardness, poor elasticity, and barely complete shape.	7–13
	Too hard, inelastic, and incomplete in shape.	0–6

**Table 2:** Sensory evaluation of marinated tofu from different brands.

	Color	Taste	Flavour	Texture	Total Score
TF1	19.00 ± 2.80 ^a^	19.83 ± 1.70 ^a^	20.42 ± 1.44 ^a^	19.17 ± 3.16 ^a^	78.42 ± 5.37 ^a^
TF2	19.25 ± 1.66 ^a^	21.17 ± 1.40 ^a^	20.75 ± 1.86 ^a^	20.17 ± 1.75 ^a^	81.33 ± 4.52 ^a^
TF3	20.75 ± 2.26 ^a^	19.67 ± 2.50 ^a^	19.58 ± 1.38 ^a^	19.58 ± 2.54 ^a^	79.58 ± 5.30 ^a^
TF4	9.42 ± 3.40 ^b^	16.08 ± 1.78 ^b^	13.92 ± 1.78 ^b^	18.08 ± 2.35 ^a^	57.50 ± 3.50 ^b^

Note: Different letters in the same column indicate a statistically significant difference (*p* < 0.05).

**Table 3:** Results of the texture determination of marinated tofu from different brands.

	Hardness(g)	Resilience	Cohesiveness	Springiness	Gumminess	Chewiness
TF1	1375.00 ± 82.50 ^c^	0.38 ± 0.01 ^b^	0.71 ± 0.01 ^b^	4.90 ± 0.23 ^b^	998.00 ± 28.60 ^d^	4809.75 ± 359.52 ^b^
TF2	1561.75 ± 90.82 ^b^	0.38 ± 0.01 ^b^	0.70 ± 0.02 ^b^	4.48 ± 0.26 ^c^	1154.25 ± 93.13 ^c^	4810.75 ± 594.78 ^b^
TF3	1655.88 ± 31.54 ^b^	0.40 ± 0.02 ^a^	0.73 ± 0.01 ^a^	3.60 ± 0.14 ^d^	1280.50 ± 70.38 ^b^	4417.75 ± 273.01 ^b^
TF4	3963.13 ± 179.44 ^a^	0.36 ± 0.01 ^b^	0.74 ± 0.01 ^a^	6.15 ± 0.23 ^a^	2869.75 ± 75.25 ^a^	17884.00 ± 750.77 ^a^

Note: Different letters in the same column indicate a statistically significant difference (*p* < 0.05).

**Table 5:** ROAV of marinated tofu from different brands.

Count	Odor Threshold μg/kg	Compound Name	ROAV			
			TF1	TF2	TF3	TF4
1	41	*α*-Pinene	2.34	2.57	2.49	2.22
2	140	*β*-Pinene	0.30	0.33	0.34	0.12
3	-	*β*-Thujene	-	-	-	-
4	770	*δ*-3-Carene	0.04	0.06	0.04	0.03
5	1.2	*β*-Myrcene	100.00	100.00	100.00	100.00
6	40	*α*-Phellandrene	1.28	1.18	1.87	0.53
7	10	(+)-Limonene	8.52	7.01	7.02	8.47
8	36	*β*-Phellandrene	2.79	2.45	1.89	2.66
9	34	*β*-Ocimene	2.92	3.61	4.00	0.77
10	1000	*γ*-Terpinene	0.02	0.02	0.03	0.02
11	200	*α*-Terpinolene	0.14	0.19	0.19	0.09
12	8200	2-Methyl-2-propanol	0.01	0.01	0.01	0.01
13	950,000	Ethanol	0.00	0.00	0.00	0.00
14	9000	1-Propanol	0.00	0.00	0.00	0.00
15	459.2	1-Butanol	0.15	0.13	0.03	0.06
16	358.1	1-Penten-3-ol	0.24	0.18	0.16	0.15
17	4	1-Butanol, 3-methyl	6.23	5.37	3.88	11.13
18	150.2	1-Pentanol	1.66	1.52	1.39	1.02
19	547.125	3-Methyl-3-buten-1-ol	0.01	0.01	0.01	0.02
20	110	(*E*)-3-Hexen-1-ol	0.07	0.06	0.05	1.12
21	5.6	1-Hexanol	5.65	6.63	6.37	17.53
22	6	Linalool	92.77	78.46	66.20	35.12
23	40,000	2-Propanone	0.02	0.02	0.02	0.02
24	35,400.2	2-Butanone	0.01	0.01	0.01	0.01
25	240	4-Methyl-2-pentanone	0.00	0.00	0.00	0.01
26	23	1-Penten-3-one	0.59	0.52	1.18	0.17
27	41	3-Hexanone	0.85	0.55	0.66	1.29
28	140	2-Heptanone	2.01	1.61	1.38	0.77
29	-	2-Methyl-3-ketotetrahydrofuran	-	-	-	-
30	14	2-Butanone, 3-hydroxy	1.65	1.55	1.99	0.93
31	10,000	1-Hydroxy-2-propanone	0.01	0.01	0.01	0.00
32	68	2-Methyl-2-hepten-6-one	0.08	0.11	0.14	0.08
33	15.1	Propanal	8.17	6.82	6.78	7.18
34	2	Butanal	4.80	1.47	3.58	5.97
35	1.1	3-Methyl butanal	78.41	46.55	70.36	75.68
36	12	n-Pentanal	19.27	15.09	15.25	21.72
37	0.3	(*E*)-2-butenal	21.27	21.03	20.61	15.14
38	4.5	1-Hexanal	62.71	54.35	51.11	77.33
39	-	(*Z*)-2-Pentenal	-	-	-	-
40	980	(*E*)-2-Pentenal	0.12	0.10	0.11	0.08
41	2.8	Heptaldehyde	7.37	6.18	6.51	14.63
42	17	(*E*)-2-Hexen-1-al	1.52	1.57	1.06	6.31
43	0.7	1-Octanal	44.62	46.26	38.56	65.71
44	13	(*E*)-2-Heptenal	0.66	0.68	0.57	4.77
45	1.1	1-Nonanal	18.32	16.80	15.99	28.52
46	350	Benzaldehyde	0.35	0.28	0.31	0.22
47	500	5-Methyl furfural	0.20	0.15	0.28	0.24
48	5	Acetic acid ethyl ester	8.69	45.23	7.18	10.87
49	1700	Isopropyl acetate	0.01	0.00	0.01	0.00
50	2000	Acetic acid propyl ester	0.06	0.01	0.02	0.01
51	66	Butyl acetate	0.18	0.57	0.20	0.05
52	22,000	Acetic acid	0.01	0.01	0.01	0.01
53	-	2,5-Dimethylfuran	-	-	-	-
54	-	Ethyl thiolacetate	-	-	-	-
55	5	2-Butylfuran	5.41	3.81	4.30	8.42
56	1.1	1,8-Cineol	16.14	16.20	51.51	16.99
57	5.8	2-Pentyl furan	87.13	70.33	58.37	46.45
58	10	Methyl thiocyanate	1.67	1.75	1.75	35.25
59	-	Methyl 2-propenyl disulfide	-	-	-	-
60	1750	2,5-Dimethylpyrazine	0.02	0.02	0.02	0.01
61	350.12	2,3,5-Trimethylpyrazine	0.04	0.03	0.06	0.03
62	-	Dipropyl disulfide	-	-	-	-
63	50	2,3-Diethylpyrazine	0.48	0.34	0.79	0.12
64	30	Allyl disulfide	0.10	0.09	0.92	0.11

## Data Availability

The original contributions presented in the study are included in the article, further inquiries can be directed to the corresponding author.
